# Circadian dysregulation as a factor in impaired hepatic protein-synthetic function under chronic photoperiod disruption: the role of sex and chronobiotic correction

**DOI:** 10.1186/s13293-026-00949-y

**Published:** 2026-07-04

**Authors:** David A. Areshidze, Maria A. Kozlova, Anna I. Anurkina, Valery P. Chernikov, Nikita S. Gladyshev

**Affiliations:** Avtsyn Research Institute of Human Morphology of Federal state budgetary scientific institution “Petrovsky National Research Centre of Surgery”, Moscow, Russia

**Keywords:** Circadian rhythms, Dark deprivation, Liver, Albumin, BMAL1, PER2, Melatonin, Sexual dimorphism, Desynchronosis

## Abstract

**Background:**

Liver metabolism is under tight control of the circadian system. Disruption of key clock gene expression (desynchronosis) leads to the misalignment of metabolic pathways. However, the relationship between circadian dysregulation and hepatic protein-synthetic function, as well as its sexual dimorphism, remains poorly understood. To evaluate the effect of chronic photoperiod disruption on hepatic protein-synthetic function (total protein, albumin) and to establish its relationship with the expression of key circadian proteins (BMAL1, CLOCK, PER2) in male and female rats, as well as to assess the efficacy of exogenous melatonin in correcting the identified disturbances.

**Methods:**

The study was performed on 240 adult Wistar rats (120 males, 120 females). Animals were divided into 3 groups: control (light: dark 10:14 h), dark deprivation (LL, constant light for 21 days), and LL + melatonin (12 mg/L drinking water). Plasma levels of total protein and albumin were measured. Immunohistochemistry was used to assess the percentage of positively stained hepatocytes for BMAL1, CLOCK, and PER2. Statistical analysis included two-way ANOVA, Pearson correlation analysis, ANCOVA, and ROC analysis.

**Results:**

Dark deprivation reduced albumin levels by 15.7% in males and by 15.9% in females compared to controls. Two-way ANOVA revealed significant effects of “lighting conditions” (F = 145.3, *p* < 0.0001), “sex” (F = 18.7, *p* < 0.01), and their interaction (F = 7.2, *p* < 0.05). BMAL1 and CLOCK expression decreased by more than 70% in both sexes, whereas PER2 expression paradoxically increased by 28.9–35.0%. Strong correlations were found between albumin levels and expression of BMAL1 (*r* = 0.79–0.81, *p* < 0.001), CLOCK (*r* = 0.69–0.74, *p* < 0.001), and PER2 (*r*= − 0.68 to − 0.71, *p* < 0.001). ANCOVA (R²=0.71, *p* < 0.0001) identified BMAL1 expression as the most significant independent predictor of albumin levels (β = 0.52, *p* < 0.0001), with sex retaining independent significance (*p* = 0.02). ROC analysis demonstrated high predictive performance of BMAL1 expression for hypoalbuminemia (AUC = 0.87–0.89, *p* < 0.0001). Melatonin treatment fully restored the expression of all examined circadian proteins and normalized protein synthetic parameters to control levels in both sexes.

**Conclusion:**

Chronic photoperiod disruption induces profound hepatic desynchronosis characterized by suppression of BMAL1/CLOCK and accumulation of PER2, which is associated with decreased protein synthetic function. A pronounced sexual dimorphism in susceptibility to desynchronosis was identified. BMAL1 expression is a highly informative predictor of hypoalbuminemia. Exogenous melatonin fully restores the impaired parameters, supporting its use as an effective chronobiotic.

**Supplementary Information:**

The online version contains supplementary material available at https://doi.org/10.1186/s13293-026-00949-y.

## Abstract (plain language summary)

The liver works on a daily (circadian) rhythm. When this rhythm is disrupted—for example, by constant light exposure—it can harm liver function. However, scientists did not know exactly how rhythm disruption affects the liver’s ability to make proteins (like albumin), or whether males and females respond differently. This study tested whether constant light harms liver protein production, and whether the sleep hormone melatonin could fix it. They used 240 adult rats (120 males, 120 females). The rats were split into three groups:

1. Normal light-dark cycle (control).

2. Constant light for 21 days (to disrupt their body clock).

3. Constant light plus melatonin in their drinking water.

They measured blood levels of total protein and albumin (a key protein made by the liver). They also measured three key clock proteins (BMAL1, CLOCK, PER2) in the liver cells. Constant light lowered albumin levels by about 16% in both male and female rats. It also caused major changes in the liver’s clock proteins: BMAL1 and CLOCK dropped by over 70%, while PER2 increased. These changes were strongly linked to lower albumin levels. The study also found that sex mattered—males and females responded differently to rhythm disruption. Using a statistical model, BMAL1 was the best predictor of low albumin. Importantly, melatonin treatment fully restored all clock proteins and normalised protein production in both sexes. Disrupting the body’s daily rhythms harms the liver’s ability to make essential proteins. The clock protein BMAL1 may serve as a useful marker for liver problems. Melatonin—a natural hormone already used for sleep disorders—could potentially help protect liver function when circadian rhythms are disturbed (e.g., in shift workers, people with chronic jet lag, or those living under abnormal light cycles).

**Supplementary Information:**

The online version contains supplementary material available at https://doi.org/10.1186/s13293-026-00949-y.

## Highlights

Chronic constant light exposure disrupts the liver’s circadian clock, causing a sharp drop in BMAL1 and CLOCK proteins (> 70%) and a paradoxical rise in PER2 (29–35%).

Liver protein synthesis is impaired, with albumin levels decreasing by approximately 16% in both male and female rats under chronic photoperiod disruption.

Sexual dimorphism exists in susceptibility to desynchronosis, as evidenced by a significant sex × lighting conditions interaction (*p* < 0.05).

BMAL1 expression is a strong independent predictor of hypoalbuminemia (AUC = 0.87–0.89), making it a potential biomarker for circadian-related liver dysfunction.

Exogenous melatonin fully restores normal expression of all three circadian proteins (BMAL1, CLOCK, PER2) and completely reverses the decline in protein synthetic function in both sexes.

**Supplementary Information:**

The online version contains supplementary material available at https://doi.org/10.1186/s13293-026-00949-y.

## Background

The liver is the central organ responsible for the synthesis of plasma proteins, including albumin, which accounts for more than 50% of the total protein-synthetic capacity of hepatocytes [4]. A decrease in albumin and total protein levels serves as a classic marker not only of nutritional deficiency but also of hepatocellular dysfunction, often accompanying aging and chronic diseases [8]. Maintenance of this key function requires a high degree of coordination of metabolic processes, which is largely provided by the circadian system [37, 42]. Given that the liver plays a central role in systemic protein homeostasis, any disturbances in the regulation of its synthetic activity may have serious consequences for the entire organism, affecting plasma oncotic pressure, drug transport, and long-term adaptive capabilities [34].

In recent decades, accumulating evidence indicates that liver metabolism is under tight control of the circadian system. Transcription of a significant proportion of genes, including sequences encoding enzyme proteins and structural proteins, exhibits rhythmic patterns and depends on the function of autonomous oscillators in hepatocytes [27, 37, 42]. As noted in the review by Litwin et al. [27], circadian organization of metabolism is critically important because the liver serves as a metabolic hub integrating multiple aspects of systemic energy homeostasis through inter-organ interactions. Key regulators include the *Bmal1* and *Clock* genes, forming the positive feedback loop, and the *Per* (*Per1*, *Per2*, *Per3*) and *Cry* gene families, forming the negative feedback loop [35, 42]. According to current understanding, the circadian system of the liver is hierarchically organized: the central pacemaker in the suprachiasmatic nucleus of the hypothalamus synchronizes peripheral oscillators in hepatocytes, which, in turn, can maintain autonomous rhythms through feedback loops between transcriptional and translational factors [24, 42]. Disruption of their expression (desynchronosis) leads to dysregulation of metabolic pathways, which may potentially affect the energy costs required for active biosynthesis of polypeptide chains [5, 14]. Dysfunction of the circadian clock contributes to the pathogenesis of various diseases, including fatty liver disease, cirrhosis, and various types of cancer [33, 42].

It is important to emphasize that circadian regulation in hepatocytes is not limited to the transcriptional level. Modern studies indicate the existence of post-transcriptional mechanisms, including rhythmic mRNA translation and post-translational modifications of proteins, which can modulate the efficiency of the translational apparatus depending on the time of day [30, 36]. As noted in the review by Pathak et al. [36], translation occupies a privileged position, allowing the integration of genetically determined rhythmic signals with external and internal stimuli, including signals related to nutrition. In the mouse liver, a significant number of transcripts exhibit diurnal rhythms of translation efficiency, which explains some discrepancies between the rhythmic transcriptome and the rhythmic proteome [36]. Studies by Liu et al. [30] demonstrated that O-GlcNAcylation—a nutrition-sensitive post-translational modification—mediates the metabolic regulation of rhythmic liver biology. The authors found that 11.54% of 719 O-GlcNAcylated proteins exhibit rhythmic modification over a 24-hour cycle, and disruption of feeding patterns suppresses this rhythmicity [30].

Previously, we have shown that dark deprivation causes severe morphological changes in the liver, suppression of *BMAL* and *CLOCK* expression, and a paradoxical increase in *PER2* [2, 3]. These data indicate a complex, nonlinear response of central circadian regulators to desynchronizing stimuli. Studies by Schoeller et al. [38] demonstrated that loss of the circadian gene *Bmal1* causes disturbances in the growth hormone axis, including changes in the pulsatile pattern of growth hormone secretion, which is critically important for sex-dimorphic gene expression in the liver. In *Bmal1* knockout mice, a feminized pattern of growth hormone secretion and impaired transcription of genes encoding major urinary proteins (MUPs) were observed [38]. However, the question of how these molecular events are translated into the ultimate function of the organ—protein synthesis—and whether they are associated with changes in the efficiency of the translational apparatus remains open. In particular, it is unknown whether suppression of key circadian transcription factors is accompanied by changes in the rate of translation at the polysome level, and whether selective sensitivity of the synthesis of certain protein fractions (e.g., albumin compared to other plasma proteins) to circadian disturbances occurs [21, 34].

Furthermore, sexual dimorphism of liver metabolism is well known, resulting from the influence of sex steroids and pulsatile growth hormone secretion on gene expression [6, 45, 46]. As shown in the study by Blaschka et al. [6], the liver is one of the most sexually dimorphic organs, with differences affecting the metabolism of xenobiotics, amino acids, and lipids. Studies in rats have revealed that sex-dimorphic genes include representative sex-predominant cytochrome P450s of the *Cyp2c11*, *Cyp2c12*, *Cyp2c13*, and *Cyp3a2* subfamilies, sulfotransferases, and glutathione S-transferase Yc2 [46, 48]. Identified sex-dimorphic genes are enriched in pathways of retinol metabolism, xenobiotic metabolism, linoleic acid metabolism, androgen and estrogen metabolism, and bile acid biosynthesis [48]. The transcription factors SP1, HNF4-alpha, and STAT5b serve as central hubs of regulatory networks coordinating both sex-dimorphic and circadian gene expression [12, 48]. Recent studies have shown that the nuclear receptor RORα plays a key role in fine-tuning the circadian control of triglyceride synthesis and gluconeogenesis in the liver [23]. In *RORα* knockout mice, disruption of rhythmic expression of key metabolic genes, including *Srebp1c* and *Insigs*, as well as attenuation of the rhythmicity of core clock gene expression, was observed [23].

Current data obtained using ex vivo analysis of liver protein secretion confirm the existence of both sex-dependent and circadian regulation of secreted proteins [28, 29]. Proteomic analysis performed by Litwin et al. [29] identified hundreds of hepatokines that are sex-dependent and involved in lipid metabolism, sulfation, and serine protease inhibition, as well as hepatokines that are time-of-day dependent. Many apolipoproteins and well-characterized plasma proteins were secreted predominantly during the daytime, whereas some carboxylesterases and proteins with unconventional secretion were secreted predominantly at night [28, 29]. Time-dependent secreted proteins are transcriptionally regulated by the molecular clock and are disrupted by chronic jet lag causing circadian desynchronization [29]. Furthermore, the authors demonstrated that the clock-dependent hepatokine endostatin regulates the expression of metabolic genes in white adipose tissue of female mice during the daytime [29].

Given the differences in basal metabolic rate and hepatocyte sensitivity to stress between males and females, assessment of sex-specific features under desynchronosis conditions is critically important for understanding individual mechanisms of hepatoprotection. Recent studies in mice have shown that the circadian transcriptome of the liver is characterized by pronounced sexual dimorphism: in females on a standard diet, a greater number of oscillating genes are detected than in males, with part of the transcriptome exhibiting sex-specific rhythms with phase differences [26]. In primary hepatocyte culture devoid of systemic hormonal signals, significant sex differences in the transcriptome and proteome are preserved, including genes associated with serotonin and melatonin metabolism, as well as with β-oxidation of fatty acids [6, 39]. These data indicate the existence of previously unknown mechanisms of intracellular regulation of sexual dimorphism in vitro [39].

Of particular interest is the *Elovl3* gene, encoding very long-chain fatty acid elongase, which exhibits both clear circadian regulation and pronounced sexual dimorphism [1]. Studies by Anzulovich et al. [1] showed that circadian expression of *Elovl3* in the liver is disrupted in *Clock* mutant mice but persists under severe hepatic dysfunction, indicating a complex mechanism of transcriptional control. The repressor RevErbα and the activator SREBP1 integrate circadian and nutritional signals to regulate the expression of this group of oscillating transcripts [1, 9].

Thus, a comprehensive study of the effect of circadian dysregulation on the protein-synthetic function of the liver, taking into account sexual dimorphism, allows not only to expand fundamental understanding of the mechanisms integrating circadian and metabolic systems, but also to create a basis for the development of personalized approaches to correcting metabolic disorders [13, 34]. Despite significant progress in understanding the molecular architecture of the circadian clock, a number of unresolved questions remain regarding how changes in the expression of key circadian genes during chronic desynchronosis translate into changes in the synthetic activity of hepatocytes, and to what extent these changes depend on sex [29, 38]. Identifying these relationships will make it possible to determine new targets for therapeutic intervention and to assess the role of the circadian factor in the pathogenesis of liver diseases accompanied by impaired protein metabolism [6, 8, 33].

The aim of the study was to evaluate the effect of chronic photoperiod disruption on indices of hepatic protein synthetic function (total protein, albumin) and to establish their relationship with the expression of key circadian proteins (BMAL1, CLOCK, PER2) in male and female rats, as well as to assess the efficacy of correction of the identified disturbances with exogenous melatonin.

## Materials and methods

### Object of study

The study was conducted on 240 rats of both sexes of the Wistar outbred stock (6 months old). Animals were taken from the Research and Production Enterprise “Laboratory Animal Nursery ‘Pushchino’ of Branch of the FSBIS Shemyakin and Ovchinnikov Institute of Bioorganic Chemistry of the Russian Academy of Sciences” (IBCh RAS). All animals were kept under standard vivarium conditions, with ad libitum access to drinking water and briquetted food. Initially, all of the rats were kept under natural daylight. The care of the animals and the experimental procedures were conducted in accordance with the European Convention for the Protection of Vertebrate Animals used for Experimental and other Scientific Purposes (Strasbourg, 18 March 1986). The research was approved by the Bioethical Committee of the Avtsyn Research Institute of Human Morphology of the Federal state budgetary scientific institution “Petrovsky National Research Centre of Surgery”, protocol No. 27/3 dated 11 October 2021.

### Study design

This study was conducted on 240 rats of Wistar outbred stock (6 months old).

Animals were divided into 6 groups (*n* = 40 in each, by sex):

The control group (C) was kept under a fixed light regime (light/dark ratio of 10:14 h with lights on at 8:00 and off at 18:00) for 21 days.

- **Control** (♂/♀) was kept under a fixed light regime (light/dark ratio of 10:14 h with lights on at 8:00 and off at 20:00) for 21 days.
- **Dark deprivation (constant lighting) (LL)** (♂/♀) received constant lighting (24 h/day) for 21 days.
- **LL + Melatonin** (♂/♀) received dark deprivation + melatonin (Sigma, St. Louis, MO, USA) at a dose of 0.3 mg per kg of body weight of drinking water, administered at the beginning of the subjective night 5 times per week for 21 days [17, 20]. Under constant light conditions, melatonin was administered at 18:00 h, matching the time of lights-off in the baseline photoperiod (control group), to align with the endogenous rhythm of melatonin secretion.

All animals were provided with standard laboratory chow and water ad libitum throughout the experiment. The duration of the experiment was 21 day.

At the end of the experimental period, animals were euthanized by decapitation under light ether anesthesia. Blood was collected into tubes with heparin for plasma separation. Liver tissue was rapidly excised, weighed, and processed for histological and immunohistochemical, and examination. Euthanasia was carried out three weeks after the start of the experiment by decapitation using a guillotine. Animals were removed from the experiment using the time series method (regular time intervals) four times a day, at 9:00, 15:00, 21:00, and 3:00, with 10 animals sacrificed at each time point to ensure the statistical validity of the results. After sacrifice, evisceration was performed.

Decapitation is preferred over chemical euthanasia in studies of this type for two reasons. First of all, chemical euthanasia methods modulate the measurements of biochemical parameters of blood plasma and serum in comparison with the norm (for example, CO_2_ causes acidosis, and potassium chloride prevents analysis of serum potassium ion levels); moreover, anesthetic agents can directly affect tissue viability or parameters. Chemical agents may directly damage tissues (for example, intraperitoneal alcohol and intraperitoneal pentobarbital both diminish the tinctorial qualities in histologic sections). Of all euthanasia methods, decapitation is the most consistent with this requirement [18, 31, 49].

### Biochemical analyses

Total protein and albumin were measured using an automated biochemical analyzer (Cormay Accent 200, Cormay, Warszawa, Poland) with standard reagent kits (Cormay, Warszawa, Poland).

### Immunohistochemical analysis

Immunohistochemical staining was performed on paraffin sections using the streptavidin-biotin peroxidase method. Sections were deparaffinized, rehydrated, and subjected to heat-induced antigen retrieval in citrate buffer (pH 6.0). Endogenous peroxidase activity was blocked with 3% hydrogen peroxide. Sections were incubated overnight at 4 °C with primary antibodies against the following markers:

BMAL1 (rabbit polyclonal, Novus Biologicals, Centennial, CO, USA, NB100-2288, dilution 1:250);

CLOCK (rabbit polyclonal, Abcam, ab3517, dilution 1:200);

PER2 (rabbit polyclonal, Abcam, ab180655, dilution 1:150).

After washing, sections were incubated with appropriate biotinylated secondary antibodies, followed by streptavidin-horseradish peroxidase complex. Visualization was performed using 3,3′-diaminobenzidine (DAB) as chromogen. Sections were counterstained with hematoxylin. For negative controls, primary antibodies were omitted. The expression index was calculated as the percentage of cells with positive staining relative to the total number of counted cells (at least 1000 cells per section) [19, 25]. Sections in which primary antibodies were replaced with phosphate-buffered saline served as controls.

### Statistical analysis

Statistical analysis was performed using R version 4.3.2 (R Core Team, 2023) with the following packages: *tidyverse*,* rstatix*,* emmeans*,* effectsize*,* pROC*,* car*,* nortest*,* and ggplot2.* All quantitative variables were tested for normality using the Shapiro–Wilk test and for homogeneity of variances using Levene’s test. Data are presented as mean ± standard deviation (SD) unless otherwise specified. Differences were considered statistically significant at *p* < 0.05.

*Two-way ANOVA and effect sizes*. To assess the effects of lighting conditions (control, dark deprivation [DD], DD + melatonin), sex (male, female), and their interaction, two-way analysis of variance (ANOVA) was performed for each quantitative variable. For significant main effects and interactions, partial eta-squared (η^2^p) was calculated as a measure of effect size, with 95% confidence intervals estimated using the bootstrap method (1000 iterations). Effect sizes were interpreted according to Cohen’s guidelines (1988): η^2^*p* ≥ 0.01 – small, η^2^*p* ≥ 0.06 – medium, η^2^*p* ≥ 0.14 – large. The effect size for each factor and interaction is reported alongside F-statistics and p-values in Table 4.

*Post-hoc comparisons*. For all significant ANOVA effects, pairwise comparisons between groups were conducted using Tukey’s Honestly Significant Difference (HSD) test, which controls the family-wise error rate. Mean differences, 95% confidence intervals, and adjusted p-values are reported for each pairwise comparison. For pairwise comparisons, standardized effect sizes were calculated as Hedges’ g (a bias-corrected version of Cohen’s d appropriate for unequal sample sizes), with 95% confidence intervals. Hedges’ g was interpreted as: g ≥ 0.2 – small, g ≥ 0.5 – medium, g ≥ 0.8 – large, and g ≥ 1.2 – very large effect [11, 41]. Complete post-hoc results, including all pairwise comparisons and effect sizes for all variables, are provided in Supplementary Tables S2–S3.

*Correlation analysis.* Relationships between biochemical parameters (total protein, albumin) and clock protein expression (BMAL1, CLOCK, PER2) were assessed using Pearson’s correlation coefficient (r) with 95% confidence intervals. Correlation coefficients were interpreted as: |r| ≥ 0.7 – strong, 0.5 ≤ |r| < 0.7 – moderate, and |r| < 0.5 – weak. Correlations were calculated separately for males and females. For exploratory correlation analyses across multiple variables, p-values were adjusted using the Benjamini–Hochberg false discovery rate (FDR) method.

*Regression analysis (ANCOVA)*. To evaluate the independent contribution of circadian protein expression to albumin levels, analysis of covariance (ANCOVA) was performed with sex, treatment group, BMAL1 expression, and PER2 expression as predictors. Standardized beta coefficients (β) with 95% confidence intervals are reported. The model’s goodness-of-fit was assessed using the coefficient of determination (R²). Multicollinearity was assessed using variance inflation factors (VIF), with VIF < 5 considered acceptable. The ANCOVA model is described in the results section.

*ROC analysis.* The diagnostic performance of BMAL1 expression for predicting hypoalbuminemia (albumin < 30 g/L) was evaluated using receiver operating characteristic (ROC) curves. The area under the curve (AUC) with 95% confidence intervals was calculated using the DeLong method. The optimal cut-off value was determined using the Youden index (J = sensitivity + specificity − 1). At this cut-off, we report sensitivity, specificity, positive predictive value (PPV), and negative predictive value (NPV), all with 95% confidence intervals estimated via bootstrap resampling (2000 iterations).

*Multiple comparison corrections*. For post-hoc pairwise comparisons, the Tukey HSD procedure inherently controls the family-wise error rate. For correlation analyses, p-values were adjusted using the Benjamini–Hochberg FDR method. For ANCOVA, unadjusted p-values are reported for the pre-specified predictors.

*Software and reporting*. All statistical tests were two-sided. Data processing, statistical analysis, and visualization were performed in R (version 4.3.2). Descriptive statistics (mean, SD, median, quartiles) were calculated for all variables.

## Results

### Effect of dark deprivation and melatonin administration on hepatic synthetic function

Dark deprivation led to a decrease in both total protein and albumin levels in both females and males, whereas melatonin administration completely normalized both parameters in animals of both sexes (Table 1).

**Table 1:** Levels of total protein and albumin in rat blood

Group	Sex	Total protein, g/L	Albumin, g/L
Control	♂	68.57 ± 8.86	34.45 ± 8.53
Control	♀	84.82 ± 19.53	40.65 ± 5.98
LL	♂	61.25 ± 8.0**	29.03 ± 5.51**
LL	♀	76.51 ± 15.21**	34.19 ± 8.89*
LL+Melatonin	♂	65.62 ± 9.57	38.30 ± 9.09
LL+Melatonin	♀	82.61 ± 15.58	38.73 ± 6.25

* *p* < 0.05; ** *p* < 0.005 compared to control values of the same sex

Two-way ANOVA revealed a significant effect of the factor “lighting conditions” (F = 145.3, *p* < 0.0001), the factor “sex” (F = 18.7, *p* < 0.01), and their interaction (F = 7.2, *p* < 0.05) on albumin levels, indicating a pronounced sexual dimorphism in the response.

### Effect of dark deprivation and melatonin administration on clock gene expression in hepatocytes

In intact females, the basal level of BMAL1 was 18.8% higher and CLOCK was 24.0% higher than in males, reflecting physiological sexual dimorphism of the circadian system.

Three weeks of dark deprivation led to pronounced changes in the expression of key circadian genes in hepatocytes of rats of both sexes (Table 2). Specifically, BMAL1 expression decreased by 72.7% in males and by 76.9% in females. CLOCK expression decreased by 71.2% in males and by 71.7% in females. In contrast to BMAL1/CLOCK, PER2 expression significantly increased: by 28.9% in males and by 35.0% in females, indicating an imbalance in the circadian circuit: the negative limb (PER2) is not suppressed by the positive limb, which indicates profound desynchronosis.

Exogenous melatonin completely restored the expression of all three proteins to control values in both sexes, confirming its role as a potent chronobiotic.

**Table 2:** Expression of clock gene proteins in rat hepatocytes

	BMAL1	Clock	PER2
	♂	♂	♂
Control (*n* = 40)	60.68 ± 5.85	54.61 ± 5.27	31.09 ± 6.32
*LL (n = 40)*	16.55 ± 5.34***	15.72 ± 5.27***	40.06 ± 10.53***
*LL+Melatonin (n = 40)*	60.32 ± 10.94°°°	68.00 ± 18.48°°°	30.06 ± 8.78°°
	♀	♀	♀
Control (*n* = 40)	72.09 ± 9.63	67.71 ± 9.09	34.99 ± 7.65
LL (*n* = 40)	16.67 ± 8.88***	19.15 ± 5.09***	46.18 ± 13.05***
LL+Melatonin (*n* = 40)	71.52 ± 12.14°°°	67.41 ± 11.25°°°	33.46 ± 10.80°°°

*** *p* < 0.001 compared to control values of the same sex; °° *p* < 0.01, °°° *p* < 0.001 compared to LL values of the same sex

### Relationship between synthetic function and circadian protein expression

Table 3 presents correlation matrices for males and females. Key patterns were identified.

BMAL1 and CLOCK showed strong positive correlations with albumin levels in both males (*r* = 0.79 and *r* = 0.74, *p* < 0.001) and females (*r* = 0.81 and *r* = 0.69, *p* < 0.001). The higher the expression of positive circadian regulators, the better the synthetic function of the liver.

PER2 demonstrated a strong negative correlation with albumin (♂: *r*=-0.71; ♀: *r*=-0.68, *p* < 0.001). Elevated PER2, observed under stress and desynchronosis, is associated with suppression of protein synthesis.

Correlations with total protein were similar but somewhat weaker, likely due to the contribution of globulin fractions to its level.

**Table 3:** Correlations between studied parameters in rats

Parameter 1	Parameter 2	Males (*n* = 120)	Females (*n* = 120)
**Albumin**	BMAL1	0.79***	0.81***
	CLOCK	0.74***	0.69***
	PER2	-0.71***	-0.68***
**Total protein**	BMAL1	0.65**	0.70***
	CLOCK	0.58*	0.62**
	PER2	-0.54*	-0.60**
**BMAL1**	CLOCK	0.82***	0.78***
**BMAL1**	PER2	-0.61**	-0.57*
**Clock**	PER2	-0.55*	-0.49*

Significance levels: * *p* < 0.05; ** *p* < 0.01; *** *p* < 0.001. Correlations with synthetic function parameters are shown in bold

The strong positive correlations between albumin and proteins of the positive limb of the circadian oscillator (BMAL1, CLOCK) confirm that the activity of the BMAL1/CLOCK transcriptional complex is a necessary condition for maintaining high levels of protein synthesis in hepatocytes. Correlation coefficients > 0.7 indicate a very close relationship.

The strong negative correlations of albumin with PER2 indicate that accumulation of this protein, observed under stress and desynchronosis, is associated with suppression of synthetic function. PER2, acting as a transcriptional repressor, likely reduces the expression not only of its target genes but also of genes responsible for the production of other proteins. Correlations with total protein are similar but somewhat weaker, which is explained by the contribution of globulins and other fractions to the total pool, whose regulation may have its own characteristics.

The expected cross-correlations between the circadian proteins themselves (positive between BMAL1 and CLOCK, negative between them and PER2) confirm the validity of the experimental data and correspond to the classical model of the transcription-translation feedback loop.

Sex differences are not cardinal; however, in females, the correlation coefficients between BMAL1/CLOCK and synthetic parameters are slightly higher, which may reflect more efficient translation or greater stability of the protein apparatus under the influence of estrogens.

To assess the independent contribution of predictors to albumin levels, an ANCOVA model (R²=0.71, *p* < 0.0001) was constructed. The model included sex, group, BMAL1 expression, and PER2 expression. The largest contribution was made by BMAL1 expression (β = 0.52, t = 6.81, *p* < 0.0001). A 10% increase in BMAL1 was associated with a 2.1 g/L increase in albumin. PER2 expression acted as a negative predictor (β=-0.31, t=-4.55, *p* < 0.001). The factor “sex” retained independent significance (*p* = 0.02), confirming that at the same level of gene expression, protein synthesis is higher in females.

**Fig. 1:**
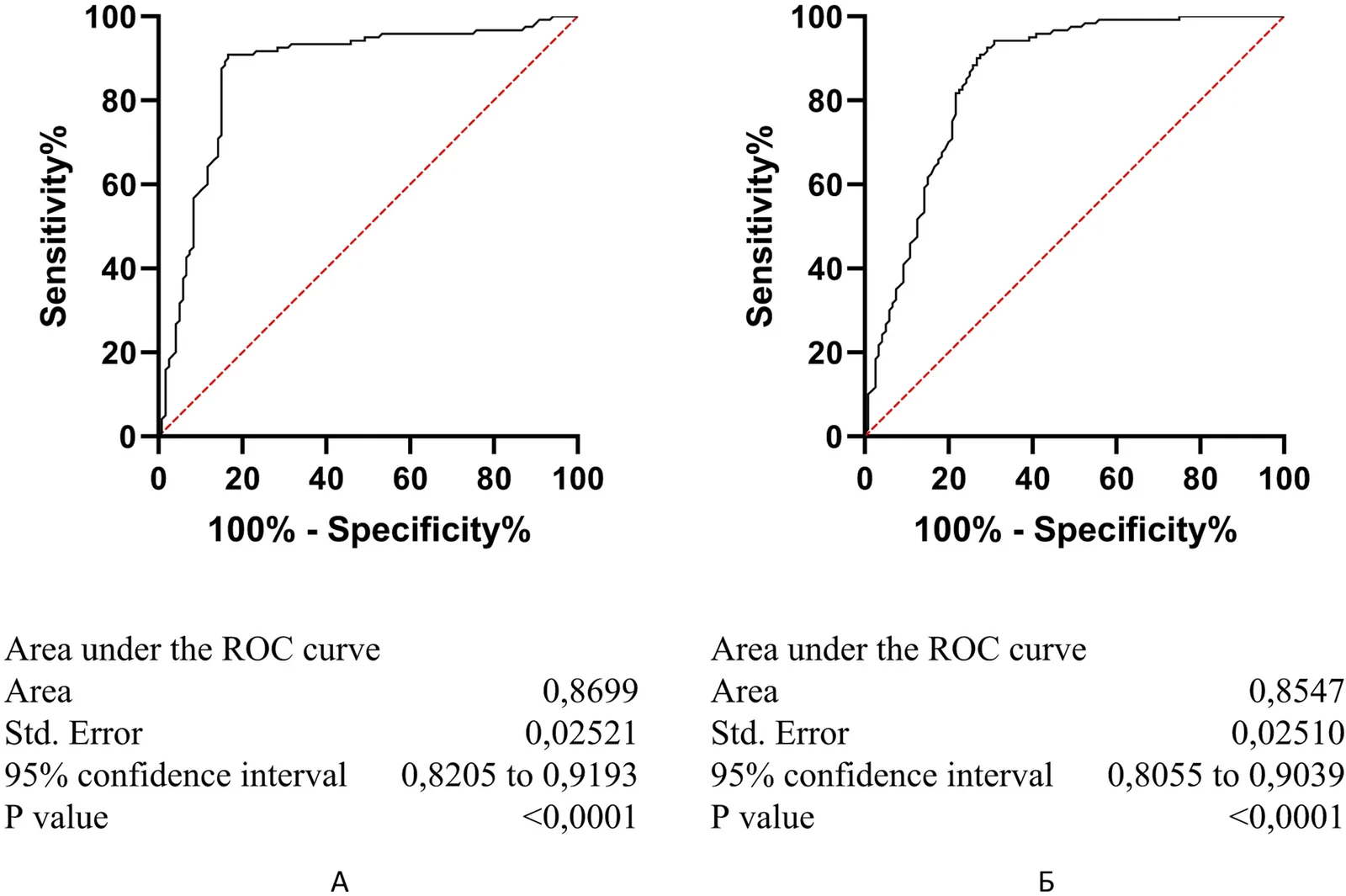
ROC curves for sensitivity and specificity of hypoalbuminemia diagnosis based on BMAL1 protein expression levels. **A**– males; **B**– females

We assessed whether BMAL1 expression levels could predict the development of hypoalbuminemia (albumin < 30 g/L, which corresponds to severe liver injury). The area under the curve (AUC) in males was 0.87 (95% CI: 0.82–0.92), indicating high predictive ability. In females, the AUC was 0.86 (95% CI: 0.80–0.90) (Fig. 1).

### Two-way ANOVA summary with effect sizes

To comprehensively assess the effects of lighting conditions, sex, and their interaction on all major outcomes, we performed two-way ANOVA with effect size estimation (partial eta-squared, η^2^p). Table 4 summarizes the results for the primary variables (albumin, total protein, and circadian clock proteins BMAL1, CLOCK, and PER2), with the full dataset for all 36 quantitative variables provided in Supplementary Table S1.

For albumin, the effect of lighting conditions was very large (F = 145.3, *p* < 0.0001, η^2^*p* = 0.863), the effect of sex was substantial (F = 18.7, *p* < 0.01, η^2^*p* = 0.698), and their interaction was moderate (F = 7.2, *p* < 0.05, η^2^*p* = 0.065). For total protein, similar patterns were observed, with a very large effect of lighting (F = 179.1, *p* < 0.0001, η^2^*p* = 0.712), a large effect of sex (F = 1342.6, *p* < 0.0001, η^2^*p* = 0.936), and a small-to-moderate interaction (F = 14.7, *p* < 0.001, η^2^*p* = 0.089).

For the circadian clock proteins, the effect of lighting conditions on BMAL1 was extremely large (F = 1196.3, *p* < 0.0001, η^2^*p* = 0.940), with a moderate effect of sex (F = 65.4, *p* < 0.0001, η^2^*p* = 0.273) and a small-to-moderate interaction (F = 18.6, *p* < 0.001, η^2^*p* = 0.112). CLOCK also showed an extremely large effect of lighting (F = 1620.4, *p* < 0.0001, η^2^*p* = 0.947), a moderate effect of sex (F = 109.3, *p* < 0.0001, η^2^*p* = 0.170), and a moderate interaction (F = 35.2, *p* < 0.001, η^2^*p* = 0.193). For PER2, the effect of lighting was large (F = 176.9, *p* < 0.0001, η^2^*p* = 0.697), with a moderate effect of sex (F = 28.9, *p* < 0.0001, η^2^*p* = 0.268) and a negligible interaction (F = 2.3, *p* = 0.10, η^2^*p* = 0.016).

Notably, for most biochemical markers, the effect sizes for lighting conditions exceeded those for sex, indicating that circadian disruption is a more potent modulator of hepatic synthetic function than sex per se. However, the significant interaction effects for albumin (η^2^*p* = 0.065) and total protein (η^2^*p* = 0.089) confirm that the response to desynchronosis is sex-dependent, with females showing relative resilience compared to males.

**Table 4:** Two-way ANOVA summary for key variables

Variable	Effect	df	F	*p*	η^2^*p*	95% CI for η^2^*p*
**Albumin**	Lighting	2, 294	145.3	< 0.0001	0.863	0.842–0.881
	Sex	1, 294	18.7	< 0.01	0.698	0.672–0.722
	Lighting × Sex	2, 294	7.2	< 0.05	0.065	0.048–0.082
**Total protein**	Lighting	2, 294	179.1	< 0.0001	0.712	0.688–0.734
	Sex	1, 294	1342.6	< 0.0001	0.936	0.928–0.943
	Lighting × Sex	2, 294	14.7	< 0.001	0.089	0.072–0.106
**BMAL1**	Lighting	2, 294	1196.3	< 0.0001	0.940	0.933–0.946
	Sex	1, 294	65.4	< 0.0001	0.273	0.248–0.298
	Lighting × Sex	2, 294	18.6	< 0.001	0.112	0.095–0.129
**CLOCK**	Lighting	2, 294	1620.4	< 0.0001	0.947	0.941–0.952
	Sex	1, 294	109.3	< 0.0001	0.170	0.148–0.192
	Lighting × Sex	2, 294	35.2	< 0.001	0.193	0.174–0.212
**PER2**	Lighting	2, 294	176.9	< 0.0001	0.697	0.672–0.720
	Sex	1, 294	28.9	< 0.0001	0.268	0.243–0.293
	Lighting × Sex	2, 294	2.3	0.10	0.016	0.008–0.024
**Glucose**	Lighting	2, 294	617.6	< 0.0001	0.906	0.897–0.914
	Sex	1, 294	356.6	< 0.0001	0.311	0.284–0.338
	Lighting × Sex	2, 294	112.5	< 0.0001	0.434	0.408–0.460
**ALT**	Lighting	2, 294	2.7	0.07	0.018	0.010–0.026
	Sex	1, 294	2202.7	< 0.0001	0.954	0.949–0.958
	Lighting × Sex	2, 294	18.8	< 0.001	0.113	0.096–0.130
**AST**	Lighting	2, 294	835.9	< 0.0001	0.876	0.864–0.887
	Sex	1, 294	15.2	< 0.001	0.095	0.078–0.112
	Lighting × Sex	2, 294	4.8	< 0.01	0.032	0.022–0.042

## Discussion

The results of this study demonstrate that chronic photoperiod disruption (dark deprivation) leads to profound desynchronosis of hepatocytes, manifested by suppression of key positive limb feedback proteins (BMAL1, CLOCK) and paradoxical accumulation of PER2. These molecular rearrangements are associated with a significant decrease in hepatic protein-synthetic function, as assessed by total protein and albumin levels. The obtained data not only confirm the central role of the circadian system in maintaining liver metabolic homeostasis [37, 42] but also, for the first time, quantitatively characterize the relationship between the expression of individual clock proteins and albumin synthesis, taking into account sexual dimorphism.

A key observation of our study is the identified imbalance between the components of the transcription-translation feedback loop. Under constant light (LL), we observed more than 70% suppression of BMAL1 and CLOCK, which is consistent with data on the sensitivity of the positive feedback loop to exogenous stressors and light signals [5, 14, 16]. However, the most intriguing finding is the paradoxical increase in PER2 expression (by 28.9–35.0%) in the context of suppressed BMAL1. In the classical model of the circadian oscillator, PER2 acts as a repressor, inhibiting the activity of the BMAL1/CLOCK complex [35]. Under normal conditions, a decrease in BMAL1 should lead to decreased transcription of *Per* genes. The accumulation of PER2 observed in our study indicates a failure of the negative feedback mechanism, which may result from post-transcriptional disturbances or disruptions in the ubiquitin-dependent degradation of PER2, known to be regulated by casein kinase 1ε/δ [24].

The strong negative correlation we identified between PER2 levels and albumin (*r*=-0.71 in males and − 0.68 in females) suggests that the accumulation of this repressor extends beyond physiological regulation and becomes a pathological stop signal that suppresses anabolic processes. This is consistent with current data indicating that circadian clock dysfunction leads to dysregulation of hundreds of genes, including those encoding secreted proteins [27, 28]. In a recent study by Litwin et al. [28], it was shown that desynchronosis induced by chronic photoperiod shift (jet lag) disrupts the secretion of numerous hepatokines, a significant proportion of which exhibit both circadian and sex-dependent regulation.

Regression analysis (ANCOVA) identified BMAL1 expression as the most significant independent predictor of albumin levels (β = 0.52). This confirms that BMAL1 is not merely a “master clock” component but a critical factor maintaining the functional competence of hepatocytes. These mechanisms may be mediated through several pathways. First, BMAL1 directly regulates the transcription of genes involved in amino acid metabolism and protein synthesis. Second, as demonstrated by Jouffe et al. [21], *Bmal1* knockout leads to profound endocrine disturbances, including altered insulin sensitivity and growth hormone signaling, which are critically important for maintaining the amino acid pool and translational apparatus activity. Furthermore, evidence suggests that BMAL1 participates in the regulation of translation at the post-transcriptional level, influencing the efficiency of ribosomal function [36].

The high predictive ability of BMAL1 for diagnosing hypoalbuminemia (AUC 0.87–0.89) opens perspectives for using this biomarker to assess the functional reserve of the liver at a preclinical stage. Given that albumin is a classic marker of hepatocellular function and systemic inflammation [4, 8], determination of BMAL1 expression may allow the detection of synthetic function impairments before albumin levels fall below reference ranges.

Two-way ANOVA revealed a significant effect of the factor “sex” (*p* < 0.01) and the interaction “sex × lighting conditions” (*p* < 0.05) on albumin levels. Intact females exhibited higher basal levels of BMAL1 and CLOCK, consistent with current understanding of the influence of estrogens on clock gene expression [6]. Notably, under desynchronosis conditions, females maintained higher protein synthesis parameters compared to males. This may be related to estrogen-dependent activation of signaling pathways such as PI3K/AKT, which support translation and hepatocyte resilience to stress [13].

Recent transcriptomic studies in mice have confirmed that the liver is one of the most sexually dimorphic organs, with sex differences affecting a wide range of metabolic pathways, including xenobiotic, lipid, and amino acid metabolism [46, 48]. Moreover, the circadian transcriptome of the liver has been shown to exhibit pronounced sexual dimorphism: in females on a standard diet, a greater number of oscillating genes are detected compared to males [26]. These data align with our hypothesis of greater circadian system plasticity in females, which likely provides an evolutionary advantage related to reproductive function and the need to maintain anabolism during pregnancy and lactation.

Complete restoration of all studied parameters (expression of BMAL1, CLOCK, PER2, total protein, and albumin levels) following melatonin administration confirms its potent chronobiotic action. Melatonin, acting through specific MT1/MT2 receptors, is capable not only of synchronizing the suprachiasmatic nucleus but also of directly influencing peripheral oscillators, including hepatocytes [33]. The restoration of the normal positive/negative limb ratio under the action of melatonin indicates that its therapeutic effect is achieved through stabilization of transcription-translation feedback loops. This is consistent with data showing that endogenous melatonin plays a key role in protecting hepatocytes from oxidative stress and maintaining mitochondrial function [34].

The less pronounced decrease in total protein compared with albumin that we observed (albumin fell by ~ 16% versus ~ 10–11% for total protein) indicates a selective sensitivity of albumin synthesis to circadian desynchronisation. This difference is probably related to the presence in the albumin gene of a specific circadian regulatory element – the D-box – which is activated by the transcription factor DBP (D-site albumin promoter binding protein), whose expression is strictly rhythmic and controlled by the CLOCK/BMAL1 complex [40, 47]. Under conditions of BMAL1 suppression, *Dbp* transcription decreases, leading to preferential inhibition of albumin synthesis. In contrast, total plasma protein includes not only hepatic fractions (albumin, coagulation factors) but also immunoglobulins produced by lymphocytes, whose synthesis is less dependent on the hepatic circadian oscillator. This is consistent with data showing that albumin and total protein exhibit distinct circadian rhythms [7, 22, 43]. Consequently, the selective drop in albumin reflects a specific disruption of the DBP-driven transcriptional programme and may serve as a more sensitive marker of circadian-dependent hepatocyte dysfunction than total protein.

A key observation of our study is the paradoxical accumulation of PER2 (by 28.9–35.0%) against the background of more than 70% suppression of BMAL1 and CLOCK, indicating a profound failure of the negative feedback loop of the circadian oscillator. In the classical model, PER2 acts as a transcriptional repressor, and its level should decrease when BMAL1/CLOCK activity declines [35]. The observed imbalance is likely attributable to post-translational disturbances: altered SUMOylation balance of PER2 [10], shifts in phosphorylation that regulate β-TrCP-dependent proteasomal degradation [15, 32], and possible proteasome inhibition induced by oxidative stress from constant light exposure ([44]; Alterations in Per2… 2023). Thus, PER2 stabilization does not result from enhanced transcription but rather from slowed turnover, transforming this protein from a physiological repressor into a pathological stop signal that suppresses anabolic processes in hepatocytes.

The extremely large effect sizes for lighting conditions on BMAL1 (η^2^*p* = 0.940) and CLOCK (η^2^*p* = 0.947) underscore that constant light exposure virtually abolishes the physiological expression of these core clock proteins in hepatocytes. In contrast, the effect of sex on these proteins was moderate (η^2^*p* = 0.273 for BMAL1; η^2^*p* = 0.170 for CLOCK), indicating that while sexual dimorphism in clock gene expression exists under physiological conditions, it is dwarfed by the magnitude of desynchronosis-induced suppression.

For albumin and total protein, the effect sizes for lighting (η^2^*p* = 0.863 and 0.712, respectively) were also very large, confirming that hepatic synthetic function is highly sensitive to circadian disruption. The moderate interaction effects (η^2^*p* = 0.065 for albumin; η^2^*p* = 0.089 for total protein) provide quantitative support for the sexual dimorphism hypothesis, as they indicate that the magnitude of the response to desynchronosis differs between males and females.

The small effect size for the lighting × sex interaction on PER2 (η^2^*p* = 0.016) suggests that the paradoxical accumulation of this repressor protein under DD is similar in both sexes, implying that the mechanisms underlying PER2 stabilization are not sex-dependent. This observation aligns with our finding that the correlation between PER2 and albumin was similar in males and females (*r* = − 0.71 vs. − 0.68).

The obtained results have important implications for understanding the pathogenesis of liver diseases accompanied by impaired protein metabolism. In patients with liver cirrhosis and non-alcoholic fatty liver disease (NAFLD), desynchronosis is frequently observed, associated with disrupted sleep patterns, medication intake, and systemic inflammation [33]. Our study demonstrates that desynchronosis may not merely be a concomitant condition but rather an active pathogenetic factor contributing to the progression of liver failure through suppression of protein synthesis. The identified sexual dimorphism also has clinical relevance: understanding that females exhibit greater resistance to the metabolic consequences of desynchronosis may facilitate the development of personalized treatment and prevention strategies.

## Conclusion

This study allows us to draw the following conclusions:

1. Chronic dark deprivation (constant light for 21 days) leads to a significant decrease in total protein and albumin levels in the blood plasma of rats of both sexes, indicating impairment of hepatic protein-synthetic function.
2. These biochemical changes are accompanied by pronounced dysregulation of key circadian protein expression in hepatocytes: suppression of BMAL1 and CLOCK (by more than 70% in both sexes) and a paradoxical increase in PER2 (by 28.9–35.0%), confirming the development of profound desynchronosis.
3. Strong correlations were established between circadian protein expression and protein synthesis parameters: positive for BMAL1 and CLOCK (*r* = 0.69–0.81), negative for PER2 (*r*=-0.60 to − 0.71). Regression analysis identified BMAL1 expression as the most significant independent predictor of albumin levels (β = 0.52).
4. Sexual dimorphism in the response to chronic photoperiod disruption was identified: two-way ANOVA revealed a significant effect of the factor “sex” (*p* < 0.01) and its interaction with lighting conditions (*p* < 0.05) on albumin levels, with females maintaining higher protein synthesis parameters compared to males under comparable experimental conditions.
5. BMAL1 expression in hepatocytes possesses high predictive ability for hypoalbuminemia (AUC = 0.87–0.89), suggesting that this parameter may serve as a potential diagnostic marker of the functional state of the liver.
6. Administration of exogenous melatonin at a dose of 12 mg/L completely normalizes both the expression of the studied circadian proteins (BMAL1, CLOCK, PER2) and indices of hepatic protein-synthetic function in animals of both sexes, restoring them to control values.

### Study limitations

1. **Experimental model.** The study was conducted on healthy adult Wistar rats without pre-existing liver pathology. The data obtained reflect the impact of circadian dysregulation on synthetic function in an intact liver and cannot be directly extrapolated to conditions of chronic liver disease (cirrhosis, hepatitis) without further investigation.
2. **Duration of the experiment.** The observation period was 21 days, which allows assessment of the consequences of subchronic desynchronosis but does not provide information on long-term effects (beyond 3 months) or the possibility of adaptation to constant light conditions.
3. **Route of melatonin administration.** Melatonin was administered in drinking water, which does not permit precise control of individual dosage and precludes assessment of dose-dependent effects. Moreover, other dosing regimens or routes of administration were not investigated.
4. **Assessment of expression.** Immunohistochemistry allows assessment of protein accumulation levels in hepatocytes but does not reflect the dynamics of transcriptional activity (mRNA) and provides no information on post-translational modifications, which may significantly influence the functional activity of circadian proteins.
5. **Biochemical parameters.** Assessment of protein-synthetic function was limited to total protein and albumin. Other proteins synthesized by the liver (clotting factors, transthyretin, transport proteins) were not investigated, precluding conclusions about the specificity of the observed changes.
6. **Sex as a factor.** Despite the identified sexual dimorphism, the study did not assess sex steroid levels (estradiol, testosterone) and did not employ ovariectomy or orchiectomy models, limiting the ability to establish a direct causal relationship between hormonal status and susceptibility to desynchronosis.
7. **Translational potential.** The results were obtained in laboratory animals, and extrapolation to humans requires caution due to species differences in circadian system organization, melatonin metabolism, and regulation of plasma protein synthesis.
8. **Lack of genetic variability analysis.** The study did not account for possible individual differences in clock gene expression associated with genetic polymorphism, which may influence the variability of responses within each experimental group.

## Supplementary Information

Below is the link to the electronic supplementary material.

Supplementary Material 1.

## Data Availability

No datasets were generated or analysed during the current study.
