# Supplementary material for: Circadian dysregulation as a factor in impaired hepatic protein-synthetic function under chronic photoperiod disruption: the role of sex and chronobiotic correction
Source: Biol Sex Differ. 2026 Jul 4;17:159. doi: 10.1186/s13293-026-00949-y (PMC13621670; doi:10.1186/s13293-026-00949-y)
Supplement: Supplementary file 1 [file 13293_2026_949_MOESM1_ESM.docx]

Table 1. Levels of total protein and albumin in rat blood.

| Group | Sex | Total protein, g/L | Albumin, g/L |
| --- | --- | --- | --- |
| Control | ♂ | 68.57±8.86 | 34.45±8.53 |
| Control | ♀ | 84.82±19.53 | 40.65±5.98 |
| LL | ♂ | 61.25±8.0** | 29.03±5.51** |
| LL | ♀ | 76.51±15.21** | 34.19±8.89* |
| LL+Melatonin | ♂ | 65.62±9.57 | 38.30±9.09 |
| LL+Melatonin | ♀ | 82.61±15.58 | 38.73±6.25 |

Note: * p<0.05; ** p<0.005 compared to control values of the same sex.

Table 2. Expression of clock gene proteins in rat hepatocytes.

|  | BMAL1 | CLOCK | PER2 |
| --- | --- | --- | --- |
|  | **♂** | **♂** | **♂** |
| Control (n=40) | 60.68±5.85 | 54.61±5.27 | 31.09±6.32 |
| *LL (n=60)* | 16.55±5.34*** | 15.72±5.27*** | 40.06±10.53*** |
| *LL+Melatonin (n=60)* | 60.32±10.94°°° | 68.00±18.48°°° | 30.06±8.78°° |
|  | **♀** | **♀** | **♀** |
| Control (n=40) | 72.09±9.63 | 67.71±9.09 | 34.99±7.65 |
| LL (n=40) | 16.67±8.88*** | 19.15±5.09*** | 46.18±13.05*** |
| LL+Melatonin (n=40) | 71.52±12.14°°° | 67.41±11.25°°° | 33.46±10.80°°° |

*Note: *** p<0.001 compared to control values of the same sex; °° p<0.01, °°° p<0.001 compared to LL values of the same sex.*

Table 3. Correlations between studied parameters in rats.

| Parameter 1 | Parameter 2 | Males (n=120) | Females (n=120) |
| --- | --- | --- | --- |
| **Albumin** | BMAL1 | 0.79*** | 0.81*** |
|  | CLOCK | 0.74*** | 0.69*** |
|  | PER2 | -0.71*** | -0.68*** |
| **Total protein** | BMAL1 | 0.65** | 0.70*** |
|  | CLOCK | 0.58* | 0.62** |
|  | PER2 | -0.54* | -0.60** |
| **BMAL1** | CLOCK | 0.82*** | 0.78*** |
| **BMAL1** | PER2 | -0.61** | -0.57* |
| **CLOCK** | PER2 | -0.55* | -0.49* |

Note: Significance levels: * p<0.05; ** p<0.01; *** p<0.001. Correlations with synthetic function parameters are shown in bold.*

|  |  |
| --- | --- |
| Area under the ROC curve \|; Area \| 0,8699; Std. Error \| 0,02521; 95% confidence interval \| 0,8205 to 0,9193; P value \| <0,0001 | Area under the ROC curve \|; Area \| 0,8547; Std. Error \| 0,02510; 95% confidence interval \| 0,8055 to 0,9039; P value \| <0,0001 |
| А | Б |

Fig. 1. ROC curves for sensitivity and specificity of hypoalbuminemia diagnosis based on BMAL1 protein expression levels. A– males; B– females.
